# ^18^F-FDG PET/CT findings of intermediate bone tumors of the spine

**DOI:** 10.1038/s41598-025-95971-2

**Published:** 2025-04-07

**Authors:** Xianwen Hu, Peqing Yang, Tengyue Mei, Jiong Cai, Pan Wang

**Affiliations:** Department of Nuclear Medicine, Affiliated Hospital of Zunyi Medical University, Zunyi, 563000 China

**Keywords:** Intermediate bone tumors, Spine, Osteoblastoma, Giant cell tumor of bone, PET/CT, Medical research, Oncology

## Abstract

Intermediate bone tumor has a certain risk of invasion and metastasis, but its invasion degree and metastasis probability are much lower than malignant bone tumor. The objective of this study was to retrospectively analyze ^18^F-FDG PET/CT findings in patients with spinal intermediate tumors. A total of 49 patients with spinal intermediate tumors consisting of 22 giant cell tumor of bone (GCTB), 11 osteoblastoma, 10 Langerhans cell histiocytosis (LCH) and 6 epithelioid hemangioma were subjected to evaluation using ^18^F-FDG PET/CT. Factors analyzed included lesion location, size, epicenter of the lesions (vertebral/posterior elements), eccentric expansile osteolysis, cortical integrity, residual bone trabeculae/spine/calcification, sclerotic rim, vertebral compression, soft tissue mass, and maximum standard uptake value (SUVmax) of lessions. GCTB, osteoblastoma, LCH, and epithelioid hemangioma showed statistically significant differences in the main body of the lesion, maximum diameter, eccentric expansile osteolysis, cortical integrity, residual bone trabeculae/spine/calcification, sclerosal margin, vertebral compression, and lesions’ SUVmax (*p* < 0.05). GCTB has a higher SUVmax, significantly higher than that of osteoblastoma and LCH (*p* < 0.05). Spinal GCTB, osteoblastoma, LCH, and epithelioid hemangioma have certain ^18^F-FDG PET/CT features, and ^18^F-FDG PET/CT may contribute to differentiate them from each other.

**Supplementary Information:**

The online version contains supplementary material available at 10.1038/s41598-025-95971-2.

## Introduction

Spinal bone tumor is one of the most common tumors in the human body, and any tumor occurring in the bone can be seen in the spine, accounting for 6–10% of bone tumors^1^. In 2013, International Agency for Research on Cancer (IARC) published the fourth revised edition of WHO Classification of Bone and Soft Tissue Tumors, edited by Fletcher and others^2^. Compared with the third edition of the classification of bone tumors, the biggest change is the addition of the category of intermediate bone tumors according to biological behavior, which is more destructive than benign bone tumors, including locally aggressive and rarely metastasizing. Locally aggressive type of intermediate bone tumors include osteoblastoma, giant cell tumor of bone (GCTB), epithelioid hemangioma, Langerhans cell histiocytosis (LCH), Erdheim-Chester disease, chondromyxoid fibroma, etc., which may recurs after surgical resection, showing invasive and locally destructive growth^3^. Rarely metastasizing type of intermediate bone tumors include GCTB, chondroblastoma, and epithelioid hemangioma, which have the ability to metastasize remotely^4^.The latest edition of the classification of bone tumors, published in 2020, has some changes on the basis of the 4th edition, which classified chondroblastoma, chondromyxoid fibroma, and aneurysmal bone cyst as benign tumors, and Erdheim-Chester disease as malignant tumors, all of which are classified intermediate tumors in the 4th edition^5,6^. Compared with benign tumors, intermediate bone tumors have a certain degree of invasion and metastasis risk, but their invasion degree and metastasis probability are far less than malignant bone tumors. Therefore, the classification methods of benign, intermediate and malignant bone tumors in the fourth and fifth editions of WHO Classification of Bone and Soft Tissue Tumors are more refined and more rigorous in dividing bone tumors according to their biological characteristics, which can better guide the formulation of clinical treatment plans. Due to the aggressiveness and metastasis of intermediate bone tumors to a certain extent, simple intra-lesion curettage, bone graft or bone cement packing has a greater local recurrence rate, and extensive curettage and extensive excision can achieve better local control and functional recovery^7^. Therefore, preoperative diagnosis is particularly important for the treatment of patients. This study retrospectively analyzed the clinical data and ^18^F-FDG PET/CT findings of intermediate bone tumors of the spine, with the aim of improving understanding of these relatively rare tumors.

## Materials and methods

### Patients

We retrospectively reviewed patients with spinal tumors who underwent ^18^F-FDG PET/CT examination between December 2019 to July 2023, and patients with pathologically confirmed intermediate bone tumors were included in this study.

The enrolled patients must meet the following criteria: (i) The patient had detailed clinical data, suspected spinal tumor after ^18^F-FDG PET/CT examination, and pathologically confirmed intermediate bone tumor after surgical treatment or needle biopsy; and (ii) the ^18^F-FDG PET/CT image of the patient is clear and will not affect the judgment of the image. However, patients who had undergone surgery, chemotherapy, radiotherapy or systemic therapies before undergoing ^18^F-FDG PET/CT examination were excluded from this study.

### ^18^F-FDG PET/CT imaging

The production and synthesis of ^18^F-FDG adopts HM-10HP cyclotron (Sumitomo Corporation, Japan) and F300E modular automatic synthesis device of FDG chemical synthesis system. When the quality control results showed that the radiochemical purity of ^18^F-FDG was greater than 95%, imaging could be performed after intravenous injection. The ^18^F-FDG-PET/CT was administered intravenously (0.1–0.15 mCi/kg) after 6 h of fasting and when blood glucose level of the patients was < 11.1 mmol/L. Imaging was performed 1 h after injection of ^18^F-FDG with Biograph mCT PET/CT scanner (Siemens, German). The scanning range was from the top of the skull to the middle of the femur or vertex to the feet depending on patient’s diagnosis and clinic. The CT scanning parameters were 120 kV tube voltage, 119 mA tube current, and the slice thickness was 5 mm. PET scan was performed immediately after the completion of CT, and the scanning parameter is 2 min per bed, with 6 to 7 beds. PET images were attenuated and corrected with CT data and reconstructed with TrueX + TOF method after image acquisition.

### ^18^F-FDG-PET/CT imaging analysis

All PET/CT images are performed by two attending nuclear medicine physicians with more than 5 years of experience in PET/CT imaging diagnosis. In case of inconsistency or disagreement, both parties shall negotiate until a consensus is reached. Factors analyzed included lesion location, size, majority of the lesions (vertebral/posterior elements), eccentric expansile osteolysis, cortical integrity, residual bone trabeculae/spine/calcification, sclerotic rim, vertebral compression, soft tissue mass, and maximum standardized uptake value (SUVmax) of lessions.

### Statistical analysis

The statistical analysis was conducted using SPSS 29.0 version (IBM Corporation, Armonk, NY). For continuous variables, normality was initially assessed using the Shapiro-Wilk test. If the data followed a normal distribution, they were presented as mean ± standard deviation, and comparisons among multiple groups were carried out using one-way ANOVA. In cases where the data did not exhibit a normal distribution, statistical description was presented as the median (Q1, Q3), and between-group comparisons were performed using the independent samples rank sum test. For count categorical data, the presentation consisted of the number of cases (%) and between-group comparisons were conducted using the chi-square test. If the conditions for the chi-square test were not met, Fisher’s exact probability method was applied. Correlation analysis was executed using Spearman’s method and the results were visualized through scatter plots. All tests were two-sided, and statistical significance was considered when *P* < 0.05.

## Results

### Patients

**Fig. 1:**
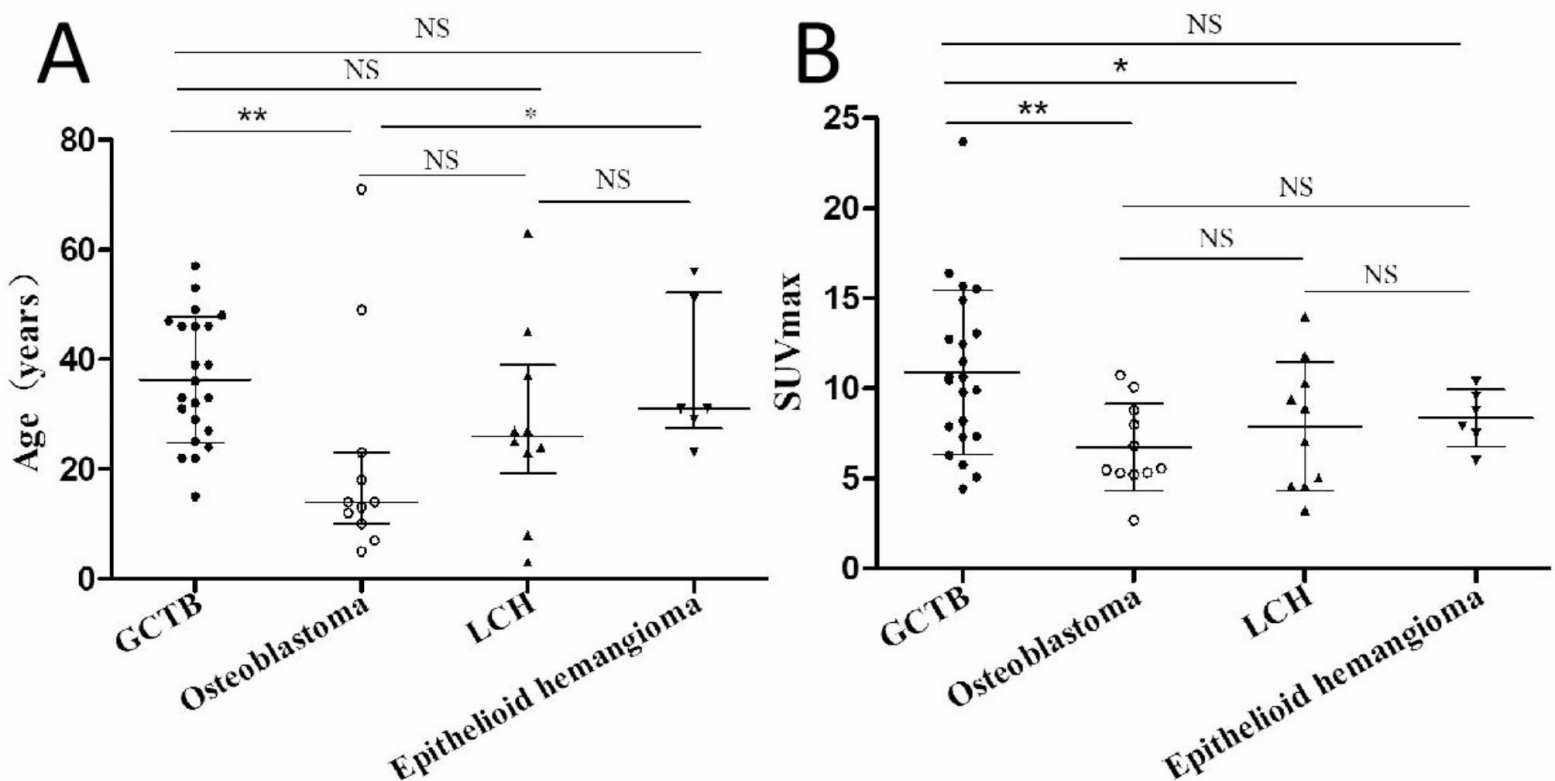
(**A**) Age distribution of patients with spinal intermediate bone tumors; (**B**) SUVmax distribution of lesions in patients with spinal intermediate bone tumors. Notes: *GCTB* giant cell tumor of bone, *LCH* langerhans cell histoplasia, *NS* no statistical significance; *p < 0.05 and **p < 0.01.

A total of 49 patients including 22 giant cell tumor of bone, 11 osteoblastoma, 10 langerhans histiocytosis and 6 epithelioid hemangioma were enrolled in the present study (Table 1). There is statistical significance in the age, among which the mean age of onset was the oldest for GCTB, which was higher than the other three intermediate bone tumors. The median age at diagnosis of osteoblastoma was lowest, and the onset age of GCTB is significantly higher than that of osteoblastoma and epithelioid hemangioma (both the p values were less than 0.05)( *p* < 0.05), as shown in Fig. 1A for details. There was no statistically significant gender difference between the tumor groups.

**Table 1:** Clinical features and ^18^F-FDG PET/CT findings of spinal intermediate bone tumors.

Parameters	GCTB (*N* = 22)	Osteoblastoma (*N* = 11)	LCH (*N* = 10)	Epithelioid hemangioma (*N* = 6)	*p*
Age	36.3 ± 11.5	14.0 [10.0, 23.0]	26.0 [19.0, 39.0]	29.0 [31.0, 59.0]	**0.006**
Gender					
Man	15 (68.2%)	7 (63.6%)	6 (60.0%)	4 (66.7%)	0.976
Woman	7 (31.8%)	4 (36.4%)	4 (40.0%)	2 (33.3%)	
Location					
Cervical vertebra	4 (18.2%)	5 (45.4%)	4 (40.0%)	0 (0.0%)	0.215
Thoracic vertebra	8 (36.4%)	1 (9.1%)	4 (40.0%)	5 (83.3%)	
Lumbar vertebra	1 (4.5%)	3 (27.3%)	2 (20.0%)	1 (16.7%)	
Sacral vertebrae	9 (40.9%)	2 (18.2%)	0 (0.0%)	0 (0.0%)	
Epicenter of the lesion					
Vertebral body	16 (72.7%)	2 (18.2%)	9 (90.0%)	4 (66.7%)	**0.002**
Posterior elements	6 (27.3%)	9 (81.8%)	1 (10.0%)	2 (33.3%)	
Maximum diameter	5.5 ± 1.6	2.4 ± 0.9	2.5 ± 0.7	3.4 ± 0.6	**< 0.001**
Cortical integrity					
Yes	4 (18.2%)	7 (63.6%)	3 (30.0%)	0 (0.0%)	**0.014**
No	18 (81.8%)	4 (36.4%)	7 (70.0%)	6 (100.0%)	
Sclerotic rim					
Yes	7 (31.8%)	8 (72.7%)	6 (60.0%)	0 (0.0%)	**0.010**
No	15 (68.2%)	3 (27.3%)	4 (40.0%)	6 (60.0%)	
Eccentric expansile osteolysis					
Yes	18 (81.8%)	10 (90.9%)	9 (90.0%)	6 (100.%)	0.656
No	4 (18.2%)	1 (9.1%)	1 (10.0%)	0 (0.0%)	
Residual bone crest/trabeculae or cacification					
Yes	18 (81.8%)	9 (81.8%)	6 (60.0%)	6 (100.0%)	0.172
No	4 (18.2%)	2 (18.2%)	4 (40.0%)	0 (0.0%)	
Vertebral compression					
Yes	14 (63.6%)	0 (0.0%)	2 (20.0%)	4 (66.7%)	**< 0.001**
No	8 (36.4%)	11 (100.0%)	8 (80.0%)	2 (33.3%)	
Soft tissue mass					
Yes	21 (95.5%)	11 (100.0%)	10 (100.0%)	6 (100.0%)	0.756
No	1 (4.5%)	0 (0.0%)	0 (0.0%)	0 (0.0%)	
SUVmax	10.9 ± 4.5	6.7 ± 2.4	7.9 ± 3.6	8.4 ± 1.6	**0.008**

*GCTB* giant-cell tumor of bone, *LCH* langerhans cell histiocytosis, *SUVmax* maximum standard uptake value.

### ^18^F-FDG-PET/CT imaging findings

The detailed ^18^F-FDG PET/CT imaging findings of patients is shown in Table 1. It is observed from the table that statistical differences were found in several different pathological types of intermediate bone tumors concerning the majority of the lesion, maximum diameter, eccentric expansile osteolysis, cortical integrity, residual bone trabeculae/spine/calcification, sclerotic rim, vertebral compression and SUVmax of lessions (all the p values were less than 0.05). Specifically, the majority of the lesion of osteoblastoma occurs in the posterior elements, while the other four intermediate tumors mainly occur in the vertebral body. For the maximum diameter of tumors, GCBTs are the largest (5.5 ± 1.6), which is larger than other tumors. In the patients included in this study, the bone cortices remained intact in most cases of osteoblastoma (7/11), while all cases of epithelioid hemangioma (6/6) and most cases of GCBT (18/22) and LCH (7/10) were incomplete. Moreover, sclerotic rims can be seen in most cases of osteoblastoma (8/11) and LCH (6/10), while all cases of epithelioid hemangioma (6/6) and most cases of GCBT (15/22) do not have this sign. Twenty-two out of 22 cases of GCTB and 4 out of 6 cases of epithelioid hemangioma had vertebral compression, whlie all cases of osteoblastoma, and most LCH (8/10) did not. As for SUVmax of lessions, GCTB is the highest, significantly higher than that of osteoblastoma and LCH (all the p values were less than 0.05), and slightly higher than that of epithelioid hemangioma, as shown in Fig. 1B.

## Discussion

### GCTB

GCTB is one of the most common primary bone tumors with potential invasive biological behavior, which is more common between the ages of 20 and 45, and there is no significant gender preference^8^. GCTB accounts for 4–5% of all bone tumors, with spinal GCTBs accounting for only 3–6% of all GCTBs^9–11^. GCTB is named after giant cells not because giant cells are true cells of tumors, but because stromal cells, the true tumor cells of GCTB, can induce the formation of multinucleated “giant cells”^10^.

**Fig. 2:**
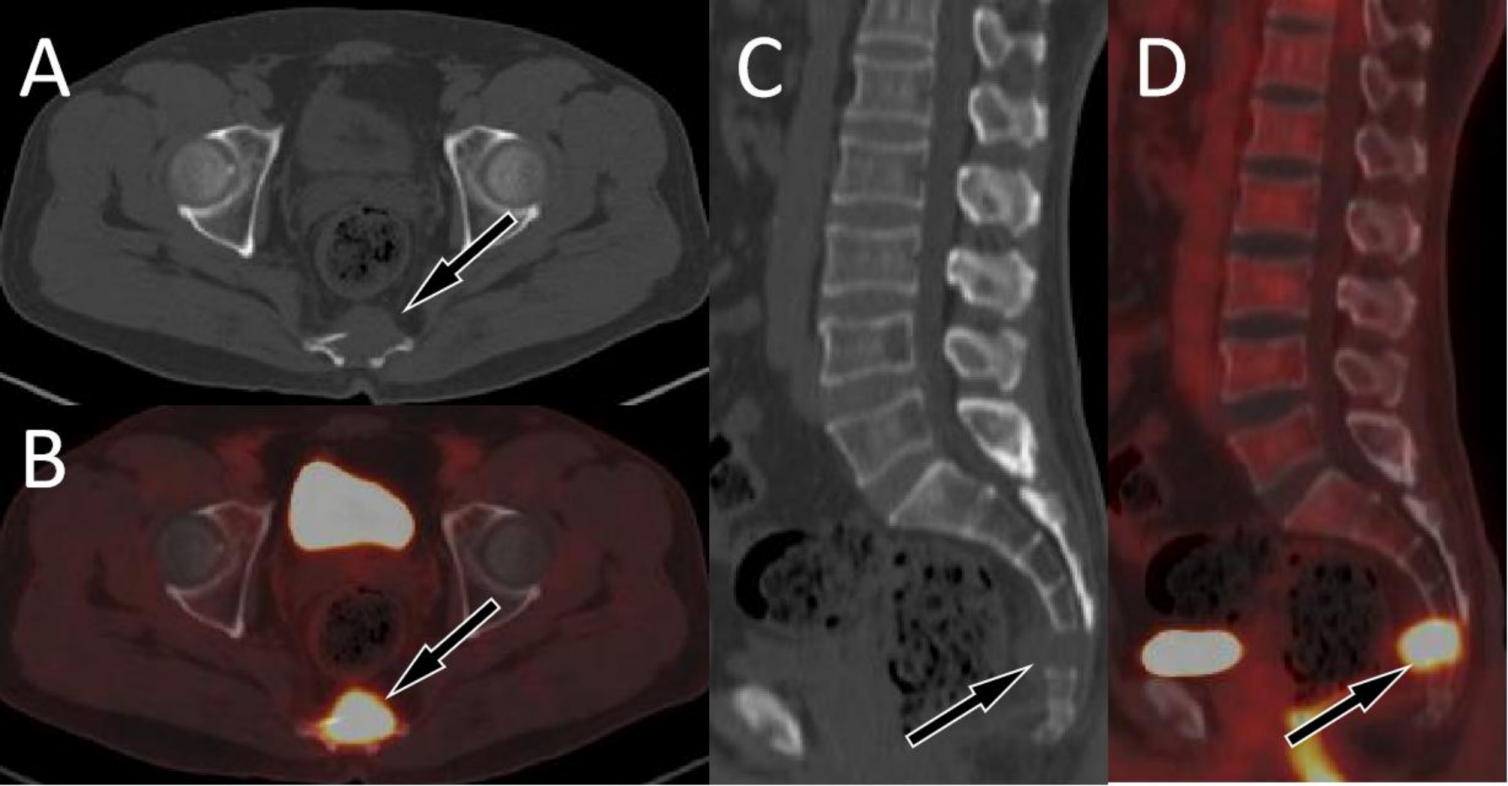
A 47-year-old woman with typical PET/CT findings of GCTB; (**A**) Axial CT shows osteolytic bone destruction in the sacral vertebrae, with slightly low-density soft tissue mass formation (arrow); (**B**) The PET/CT fusion image at the same level shows significantly increased ^18^F-FDG uptake, with a SUVmax of 12.5; Sagittal (**C** CT; **D** PET/CT) shows almost complete dissolution of the affected sacral vertebrae (arrows).

This group of cases includes 22 cases of spinal GCTB, with an average age of 36.3 ± 11.5 years old. There are more male patients than female patients, with 15 males and 7 females respectively. Most of the tumors occurred in the thoracic and sacral vertebrae, with the main body of the lesion located in the vertebral body, showing eccentric, dilatant and lytic bone destruction. Residual bone crista can be seen in it, most of them do not have sclerotic edges, calcification and periosteal reaction, and most of the lesions can break through the bone cortex to form paravertebral soft tissue mass. On PET, these lesions typically present obviously increased ^18^F-FDG uptake (as shown in Fig. 2), with an average SUVmax of 10.9 ± 4.5. Few PET/CT studies of spinal GCTB have been reported in the literature. A previous research results was consistent with our findings that GCTB showed a higher SUVmax, with a median SUVmax of 9.0 ± 2.0 ^12^. Only a few spinal GCTB lesions are mainly located in the vertebral appendages, with visible sclerotic rim and mildly increased ^18^F-FDG uptake (Fig. S1).

### Osteoblastoma

In the previous classification of bone tumors published by WHO, osteoblastoma was divided into two subtypes, namely conventional osteoblastoma and aggressive osteoblastoma^3,13^. However, in the latest version of 2020, this classification method was abandoned and it was uniformly defined as intermediate tumor^5,6^. It can occur at any age, but mainly affects children aged 10–15, characterized by its osteogenic and locally invasive behavior^14,15^. Osteoblastoma was more common in the spine, especially in the lamina and pedicle of posterior elements, accounting for approximately 10% of all spinal bone tumors^16^.

**Fig. 3:**
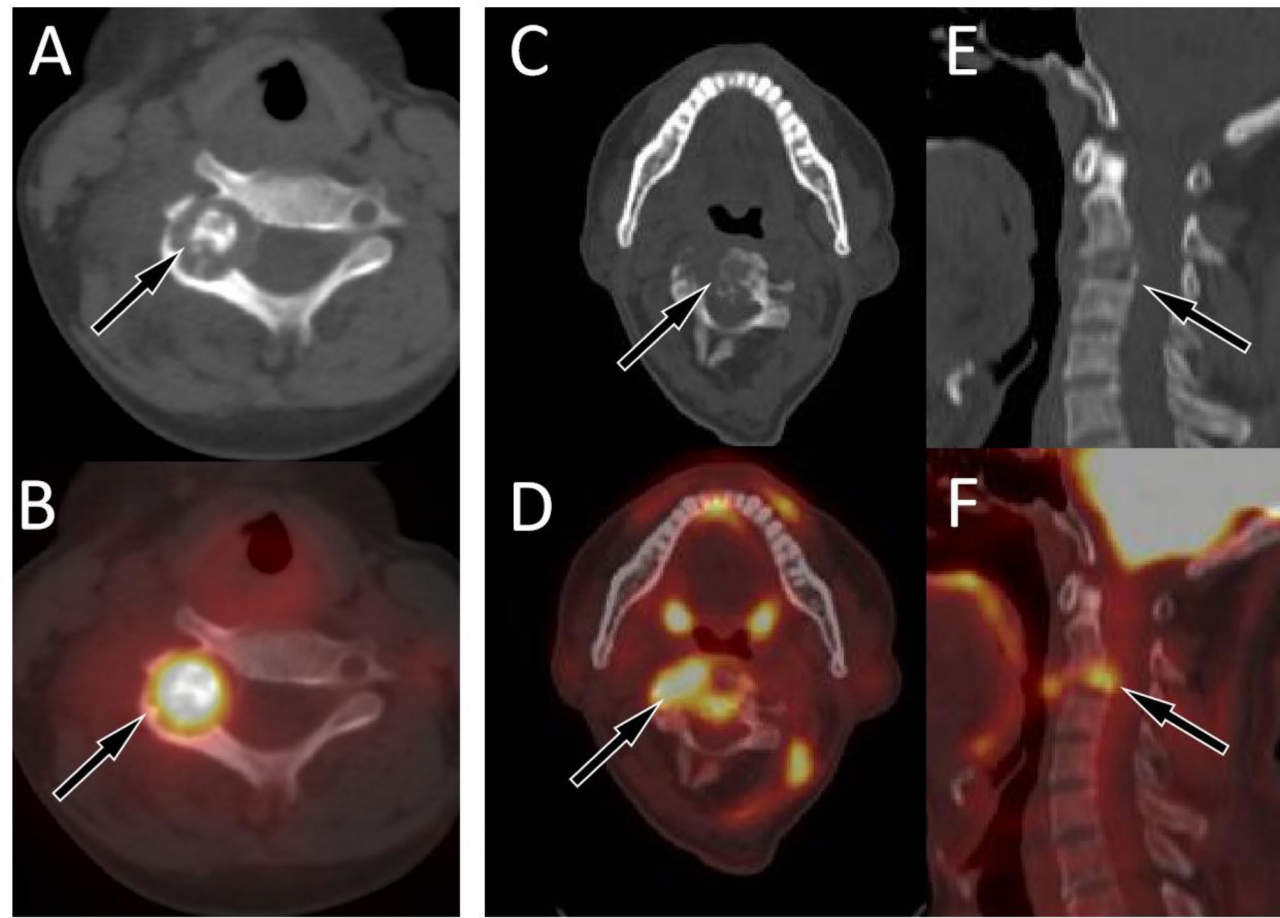
(**A**,**B**) A 10-year-old girl with typical PET/CT findings of osteoblastoma; (**A**) Axial CT revealed osteolytic bone destruction in the right vertebral plate of the fifth cervical vertebra, with nodular ossification at the center of the lesion (arrow); (**B**) The PET/CT fusion image of the corresponding area shows increased ^18^F-FDG uptake (arrow), with a SUVmax of 8.1. (**C**–**F**) A 18-year-old man with atypical PET/CT findings of osteoblastoma; (**C**) Axial CT revealed osteolytic bone destruction in the fourth cervical vertebra, with the main lesion located in the vertebral body and multiple residual bone ridges visible within the lesion (arrow); (**D**) The PET/CT fusion image of the corresponding area shows increased ^18^F-FDG uptake (arrow), with a SUVmax of 6.8; Sagittal (**E** CT; **F** PET/CT) reveals compressive changes of the vertebral body (arrows).

Our study included 11 patients with osteoblastoma originating from the spine, with the median age of onset being 14 years old. The lesions were most commonly distributed in the cervical spine, with the majority of lesions located in the posterior elements. Osteoblastoma had a small volume and can also show expansive bone destruction on CT, but the bone cortex is usually intact and accompanied by sclerotic rims. The center of the lesion usually shows residual bone ridges or characteristic high-density calcifications (Fig. 3A, B). These lesions showed an increased ^18^F-FDG uptake on PET, with a mean SUVmax of 6.7 ± 2.4, which is slightly lower than the SUVmax reported in the literature^13^. The epicenter of a few lesions may occur in the vertebral body, and accompanied by pathological compression fractures of the vertebral body (Fig. 3C–F).

### LCH

LCH is a rare disease of mononuclear phagocytosis characterized by langerhans cell overactivation, abnormal proliferation and granuloma, and the incidence rate of children is 2–5 times that of adults^17,18^. LCH can affect organs and tissues of different systems, and bone lesions are one of the main manifestations of LCH patients, of which pathological manifestations mainly include granulomas growing from the bone marrow cavity, destroying bone, and accompanied by soft tissue infiltration to form localized soft tissue masses^19^. It is mostly distributed in the vertebral body, flat bones, and long bones, most of which present osteolytic bone destruction^20^. Among them, vertebral lesions are characterized by uneven vertebral density, local cortical discontinuity, and the formation of soft tissue masses. These masses protrude into the medullary cavity and compress the spinal cord, resulting in compression symptoms^21^. Different stages of lesions present different ^18^F-FDG uptake on PET/CT, and an obviously increased ^18^F-FDG uptake indicates that the lesion is active^20,21^.

**Fig. 4:**
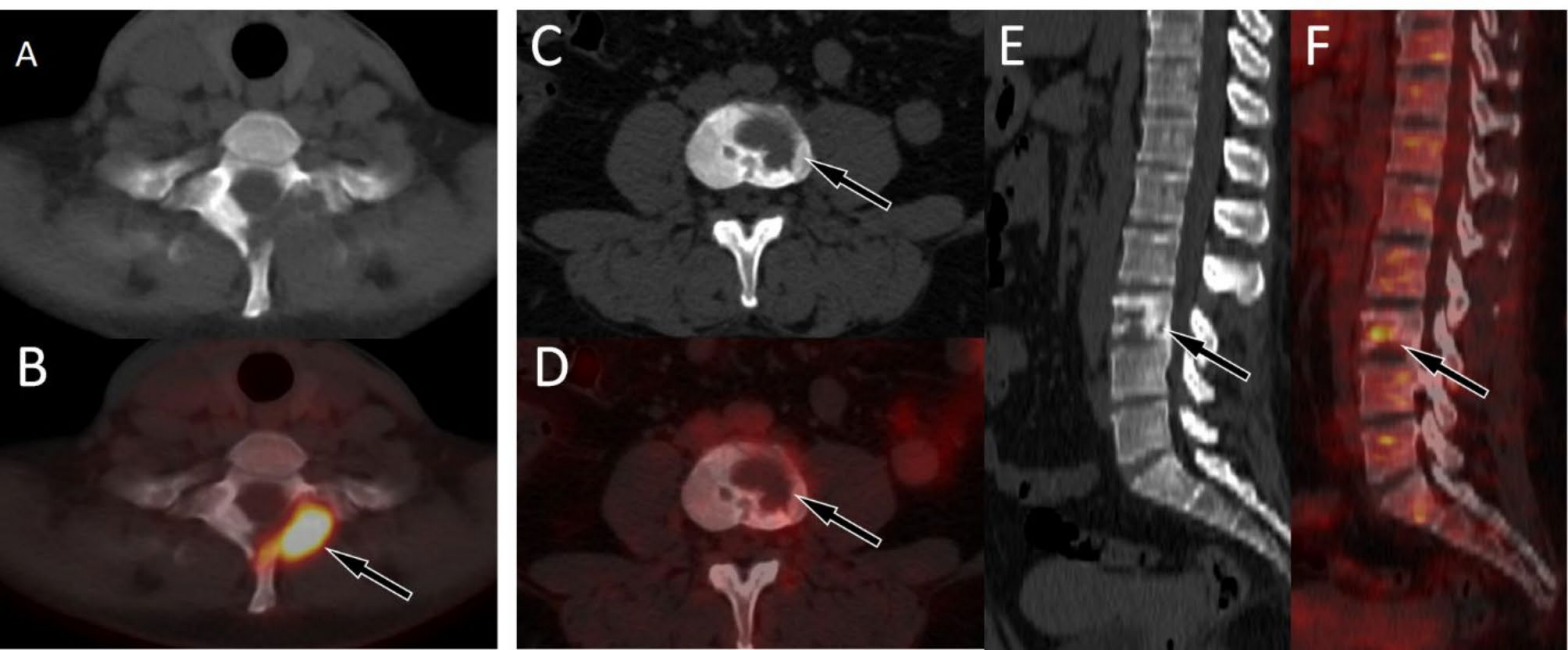
(**A**,**B**) A 37-year-old woman with LCH; (**A**) Axial CT reveals low-density osteolytic bone destruction shadow on the left vertebral plate of the first thoracic vertebra, without sclerotic rims (arrow); (**B**) The PET/CT fusion image of the corresponding area shows obviously increased ^18^F-FDG uptake (arrow), with a SUVmax of 8.9, suggesting that the lesion is in an active phase. (**C**–**F**) A 23-year-old woman with LCH; (**C**), Axial CT revealed low-density osteolytic bone destruction in the third lumbar vertebrae (arrow), without the formation of soft tissue masses; (**D**), The PET/CT fusion image of the corresponding area shows mildly increased ^18^F-FDG uptake (arrow), with a SUVmax of 3.5; and sagittal images (**E** CT; **F** PET/CT) revealed lesions with osteosclerotic rims (arrows), suggesting that the lesion may be in remission. LCH = langerhans cell histiocytosis.

Our current study enrolled a total of 10 LCH patients with spinal involvement, with a median age of 26 years old, which differs from the literature reporting that LCH is more common in children. This may be related to our inclusion of only LCH patients with spinal lesions. The epicenter of the lesions are located in the vertebral body and usually present as osteolytic destruction with incomplete bone cortex, while less causing vertebral compression. Lesions in the active phase typically do not have sclerotic rims and significantly increased ^18^F-FDG uptake (Fig. 4A, B). However, lesions in the stable phase are the opposite, usually accompanied by sclerotic rims and mildly increased ^18^F-FDG uptake (Fig. 4C–E). It is worth mentioning that none of the spinal LCH patients enrolled in our study had lesions compressing the spinal cord, which is inconsistent with what has been reported in the above literature^21^.

### Epithelioid hemangioma

Epithelioid hemangioma of bone previously classified as an intermediate locally aggressive and rarely metastasizing tumor, while in the 2020 WHO classification, it is uniformly classified as a intermediate locally aggressive tumor^5,6^. It consists of epithelioid cells and endothelial cell differentiation, and can occur at any age, but is most common between the ages of 30 and 40 ^22^. It usually involves the long tubular bones, the short tubular bones of foot, the flat bones, and the vertebrae^23,24^. It is usually a single lesion, with about 20% being multifocal^5^.

**Fig. 5:**
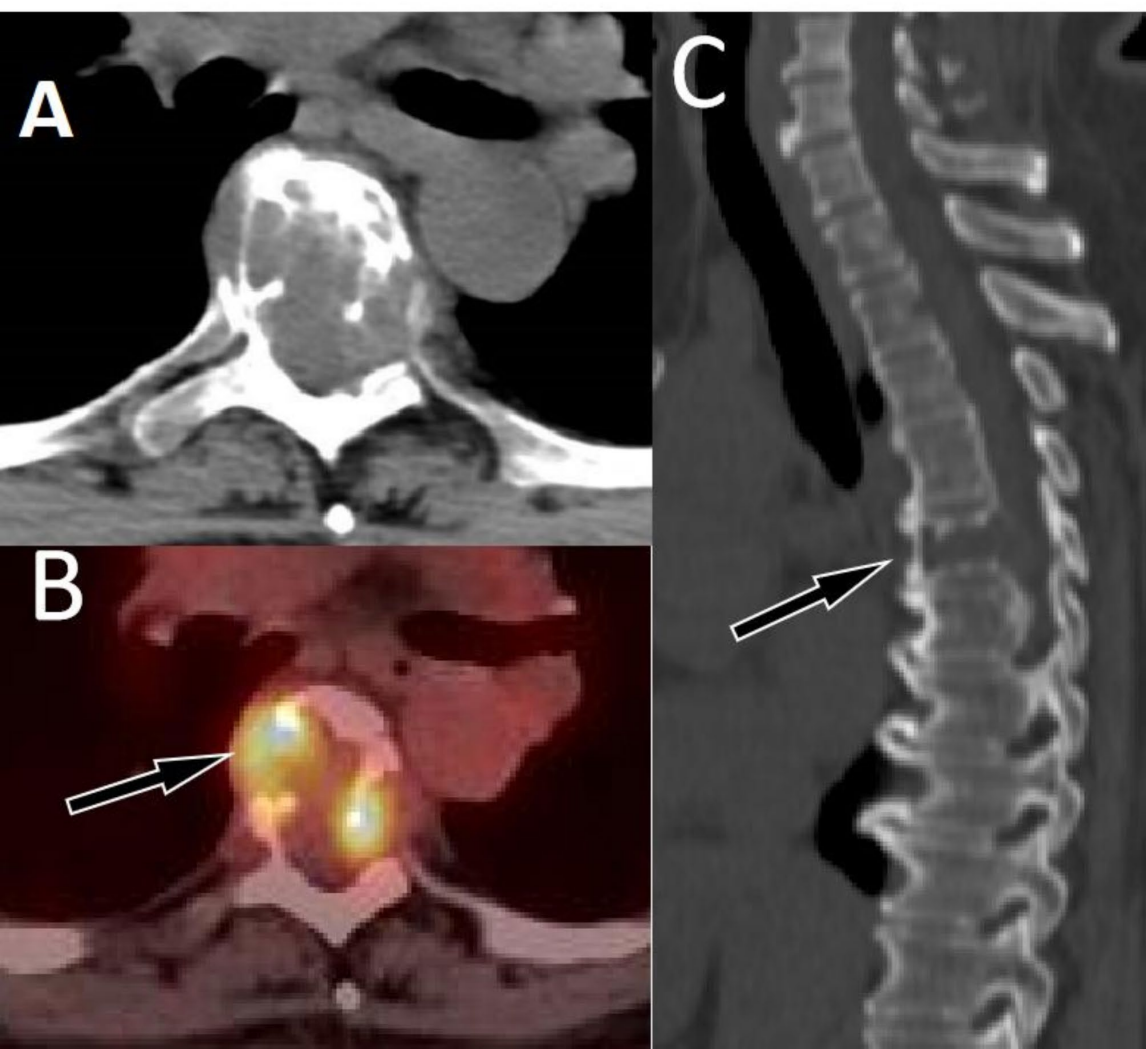
A 51-year-old woman with EHE; (**A**) Axial CT revealed low-density osteolytic bone destruction shadow in the 6th thoracic vertebral body (arrow), which broke through the bone cortex and formed a soft tissue mass in the surrounding area; (**B**) The PET/CT fusion image of the corresponding area shows mildly increased ^18^F-FDG uptake (arrow), with a SUVmax of 6.0; (**C**) Sagittal CT reveals compressive changes in the vertebral body, with low-density cavity changes in the neutral state of the vertebral body (arrow), without sclerotic rims.

The current study included a total of 6 patients with epithelioid hemangioma, most of whom were located in the thoracic vertebrae, with the main lesion located in the vertebral body, but involving the vertebral plate or/and pedicle. In this group, all lesions presented eccentric osteolytic destruction without sclerotic edges, and the lesions broke through the bone cortex and formed soft tissue mass. Moreover, residual bone trabeculae can be seen within the lesion, and most vertebral bodies show compressive changes. On PET, obviously increased ^18^F-FDG uptake by lesions was observed (Fig. 5A-C).

### Differential diagnosis

Among the four types of intermediate tumors included in this group, it is difficult to distinguish between GCTB and epithelioid hemangioma, both of which occur more frequently in the fourth decade of life. On PET/CT imaging, both present eccentric osteolytic bone destruction, mostly without sclerotic rims, accompanied by increased ^18^F-FDG uptake. However, the expansive bone destruction of GCTB is more pronounced than that of epithelioid hemangioma, with a larger lesion volume and higher SUVmax. Osteoblastoma is more common in children, and its main lesion is mostly located in posterior elements such as the vertebral plate or pedicle. On CT, the center of the lesion with osteolytic destruction is accompanied by characteristic calcification or ossification shadows, accompanied by increased ^18^F-FDG uptake. It is not difficult to differentiate it from other intermediate bone tumors. LCH can affect multiple systemic organs and tissues throughout the body, often accompanied by multiple lesions or other bone invasions when invading the spine. Depending on the activity of the lesions, it can show varying degrees of increased uptake of ^18^F-FDG on PET/CT, which can be differentiated from other tumors. The characteristics and diagnostic points of these four intermediate tumors are summarized in Table 2.

**Table 2:** Key diagnostic points of intermediate bone tumors of different pathologic types.

Tumor types	Onset age	Location	PET/CT findings	
			CT features	PET features
GCTB	Prefer middle-aged patients	More common in thoracic vertebrae and sacral vertebrae	Lesions are predominantly centered in the vertebral body, with ‌incomplete cortex‌, ‌eccentric expansile bone destruction‌ and ‌soft tissue mass‌; a sclerotic rim‌ may rarely be observed ‌‌	Obviously increased ^18^F-FDG uptake
Osteoblastoma	Prefer young patients	More common in cervical and lumbar spine	Lesions are typically centered in posterior elements, with ‌complete bone cortex; ‌soft tissue masses, ‌residual bony ridges/calcifications, and ‌sclerotic rims‌ in most cases	Increased ^18^F-FDG uptake
LCH	Prefer middle-aged patients	More common in thoracic vertebra and cervical vertebra	Lesions are ‌predominantly centered within the vertebral body, which may also exhibit ‌eccentric expansile osteolytic destruction‌ and ‌soft tissue mass; but most of the diseased vertebral bodies are not accompanied by vertebral compression, and the lesions in the stable period can present sclerotic rim.	Active lesions demonstrate increased ^18^F-FDG uptake, while stable lesions typically show no significant uptake.
Epithelioid hemangioma	Prefer middle-aged patients	More common in thoracic vertebra	Similar to GCTB, lesions are predominantly centered in the vertebral body, with ‌incomplete cortex‌, eccentric expansile bone destruction‌ and ‌soft tissue mass‌; but with rare sclerotic rim	Increased ^18^F-FDG uptake

*GCTB* giant-cell tumor of bone, *LCH* langerhans cell histiocytosis.

Moreover, intermediate tumors of the spine need to be differentiated from some malignant tumors such as osteosarcoma, chondrosarcoma, plasma cell tumor, metastatic tumor, lymphoma, and benign tumors such as osteofibrous dysplasia and bone cysts. Compared with intermediate bone tumors, osteosarcomas and high-grade chondrosarcomas may also exhibit a stronger increased uptake of ^18^F-FDG due to their high malignancy, but are usually accompanied by periosteal reactions and tumor bone formation on CT^25,26^. Plasma cell tumors that invade the spine include solitary plasma cell tumors and multiple myeloma, often presents as motheaten, punch-like, or granular low-density bone destruction, and some.

patients only show osteoporosis-like changes, with mildly to moderatly increased ^18^F-FDG uptake^27,28^. Metastatic tumors are usually accompanied by a history of malignant tumors, and patients without a history of malignant tumors can mostly detect the primary lesion on PET/CT, making differentiation easy^29^. Primary spinal lymphoma is mostly anaplastic B-cell lymphoma or diffuse large B-cell lymphoma, which presents as a larger paravertebral soft tissue mass growing around the vertebral body on CT, accompanied by strongly increased ^18^F-FDG uptake on PET^30,31^. Benign tumors or tumor like lesions that occur in the spine are relatively rare, mainly including osteofibrous dysplasia, bone cyst and so on. Osteofibrous dysplasia is characterized by well-defined uniform low-density shadows, thinning of the bone cortex, but the bone cortex is usually intact, without the formation of soft tissue masses^32^. It appears as mildly increased ^18^F-FDG uptake on PET^33^. Bone cysts present uniform low-density shadows growing longitudinally along the vertebral body, with few sclerotic rims, and there is usually no or only mildly increased ^18^F-FDG uptake on PET^25,33^.

There are limitations to the current study, first, the small sample size, retrospective design, and single-center nature of the study may limit the generalizability of the findings. Furthermore, SUVmax cutoff values were not determined for distinguishing GCTB from other tumors, which is another limitation. Therefore, the above limitations should be taken into account when applying our findings.

## Conclusion

Spinal intermediate tumors, including GCTB, osteoblastoma, LCH, and epithelioid hemangioma, have certain characteristics of ^18^F-FDG PET/CT. ^18^F-FDG PET/CT may contribute to differentiate them from each other and from other benign and malignant spinal tumors.

## Electronic supplementary material

Below is the link to the electronic supplementary material.

Supplementary Material 1

## Data Availability

The authors confirm that the data supporting the findings of this study are available within the article.

## Abbreviations

CT: Computed tomography
^18^F-FDG: Fluorine-18 fluorodeoxyglucose
PET: Positron emission tomography
GCTB: Giant cell tumor of bone
LCH: Langerhans cell histiocytosis
SUVmax: Maximum standardized uptake value
ROI: Region of interest
