# Supplementary material for: 18F-FDG PET/CT findings of intermediate bone tumors of the spine
Source: Sci Rep. 2025 Apr 7;15:11821. doi: 10.1038/s41598-025-95971-2 (PMC11977186; doi:10.1038/s41598-025-95971-2)
Supplement: Supplementary file 1 — Supplementary Material 1 [file 41598_2025_95971_MOESM1_ESM.doc]

**Supplementary materials**

**
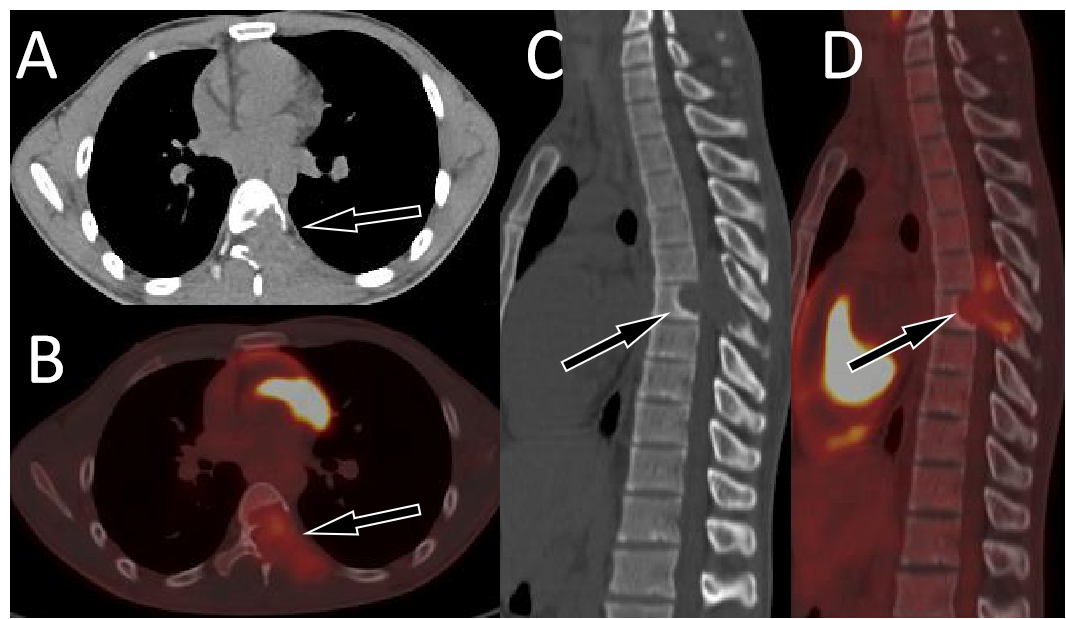
**

**Figure S1**. A 22-year-old man with atypical PET/CT findings of GCTB; **A**, Axial CT shows osteolytic bone destruction of the 7th thoracic vertebra, with the main lesion located in the left vertebral plate and pedicle, accompanied by soft tissue mass formation; **B**, The PET/CT fusion image of the corresponding area shows mildly increased ^18^F-FDG uptake, with a SUVmax of 5.1; Sagittal (**C**, CT; **D**, PET/CT) revealed sclerotic rims (arrow) at the lesion without vertebral compression. GCTB=Giant cell tumor of bone.
